# Codon Usage Bias Analysis of Citrus Leaf Blotch Virus

**DOI:** 10.3390/v17111497

**Published:** 2025-11-12

**Authors:** Xin Ren, Lifang Xu, Yuqian Yan, Ying Wang, Aijun Huang

**Affiliations:** 1 College of Life Science, Gannan Normal University, Ganzhou 341000, China; 1251016006@gnnu.edu.cn (X.R.); 1241016015@gnnu.edu.cn (L.X.); 221602038@gnnu.edu.cn (Y.Y.); wangying@gnnu.edu.cn (Y.W.); 2 National Navel Orange Engineering Research Center, Ganzhou 341000, China

**Keywords:** citrus leaf blotch virus, codon usage, host adaptation

## Abstract

Citrus leaf blotch virus (CLBV) is a positive-sense single-stranded RNA virus belonging to the genus *Citrivirus* within the family *Betaflexiviridae*. It infects a broad range of economically significant fruit crops, including citrus, kiwifruit, and apple. Surveys conducted in the field have documented appreciable incidence rates in several hosts, thereby emphasizing its emerging threat to global pomiculture. Comprehensive surveillance of CLBV genetic diversity is indispensable for predicting strain-specific epidemics and designing durable, broadly protective control strategies. Current surveys of CLBV diversity are still gene-fragment-centric, with whole-genome resolution remaining largely untapped. In this study, an analysis of codon usage bias analysis was performed using all available CLBV full-length genomes. The findings revealed that CLBV exhibits low codon usage bias, with natural selection, rather than mutational drift, being the primary driver. Phylogenetic analysis has been demonstrated to categorize isolates according to their host of origin rather than their geographical location. This observation suggests that host adaptation may supersede spatial structure in CLBV evolution and reinforce natural selection as the dominant force shaping its codon usage landscape. From the perspective of the codon adaptation index, *Prunus avium* is the host that exerts the greatest influence on the formation of its codon usage bias. The present study provides the first genome-wide portrait of CLBV codon usage bias, offering a robust framework for future investigations into its origin and evolutionary dynamics.

## 1. Introduction

Citrus leaf blotch virus (CLBV), assigned to the genus *Citrivirus* of the family *Betaflexiviridae*, has a broad natural host range encompassing citruses, kiwifruits, apples, peonies, and mulberries [1,2,3,4,5,6]. Infected plants may remain asymptomatic or display faint leaf mottling and vein clearing, depending on host genotype and environment [7,8]. Virions are flexuous filaments, about 960 × 14 nm that package an 8747 nt, positive-sense, single-stranded RNA genome containing a 3′ poly(A) tail [9,10]. Three ORFs are expressed: ORF1 yields a 227 kDa replication polyprotein harboring methyltransferase, AlkB, OTU-like peptidase, papain-like protease, helicase, and RdRp domains; ORF2 encodes a 40 kDa movement protein (MP) of the 30 K superfamily; and ORF3 specifies a 41 kDa coat protein (CP) [11]. MP and CP are translated from dedicated sub-genomic RNAs, and the MP additionally functions as a suppressor of RNA silencing [12].

CLBV remains an economically overlooked pathogen, and has not yet caused large-scale economic losses in its known hosts, so the virus has attracted only limited research interest thus far. Published investigations are largely confined to field incidence surveys and fragment-based descriptions of genetic diversity. Recent surveys of major kiwifruit and citrus production areas in China revealed CLBV detection rates of between 11.3% and 28.5% in kiwifruit and approximately 7.7% in citrus. These results indicate that the virus has already established a measurable prevalence in these economically important crops [13,14]. Phylogenetic analyses based on the movement protein (MP) and coat protein (CP) genes show minimal divergence among isolates from different geographical origins, but substantial divergence among isolates from different host species, implying that host adaptation has driven the observed variability. Because existing studies have relied almost exclusively on partial genomic sequences, comprehensive analyses using complete genome sequences are now required to provide a more robust understanding of CLBV evolution and epidemiology.

Codon usage bias (CUB) describes the non-random choice among synonymous triplets that specify the same amino acid [15]. Across all kingdoms of life, 61 sense codons are deployed with markedly unequal frequencies, generating species-specific yet genome-wide patterns that are more conserved than protein sequences themselves [16]. The observed bias can be attributed to a tripartite balance, comprising directional mutation pressure, natural selection for translational efficiency and accuracy, and genetic drift, which fixes minor alleles in small populations [17]. Additional layers, including gene length, mRNA secondary structure, protein-fold hydropathy, replication timing, and environmental stress, fine-tune local codon choice [18], whereas the tRNA gene copy number and wobble-base modification provide the ultimate selective arena [19,20,21,22]. In plant viruses these forces are amplified by an intracellular arms race: RNA viruses exhibit unusually low CUB, and adopt a “generalist” strategy to evade competition with highly expressed host photosynthetic genes. Non-recombinant isolates of the Potato virus Y display uniformly low codon preference, and every gene of the Turnip mosaic virus exhibits ENC values > 45, reducing competition with the divergent tRNA pools of their cruciferous hosts [23,24].

Despite the increasing number of CLBV genome sequences, a systematic assessment of codon usage bias as an evolutionary driver is still lacking. Here, we employed all available CLBV full-length genomes to dissect, for the first time, the codon usage landscape of citrus leaf blotch virus and to reconstruct its phylogenetic history. This combined approach offers new insight into the virus’s genetic divergence and the determinants that shape its synonymous codon choice.

## 2. Materials and Methods

### 2.1. Virus Isolates

A total of 56 full genomic sequences of CLBV isolates were downloaded from the National Center for Biotechnology Information (NCBI) (https://www.ncbi.nlm.nih.gov/, accessed on 20 June 2025). Metadata for each isolate, including date of collection, geographical location, and host plant, are provided in Table S1.

### 2.2. Recombination and Phylogenetic Analysis

The alignment of all CLBV sequences was conducted utilizing the MUSCLE method and employing the MEGA 12 software. The potential occurrence of recombination events was screened via seven algorithms (RDP, BOOTSCAN, MAXCHI, SISCAN, CHIMAERA, 3SEQ, GENECONV) within the RDP4 software suite. The assumptions regarding parent/donor assignments were confirmed through the implementation of a phylogenetic approach in the RDP4 suite. Each candidate recombinant event was corroborated by a minimum of four independent algorithms within the RDP4 suite, with *p*-values below 1.0 × 10^−6^.

The phylogenetic relationships of the three protein-coding sequences of CLBV were assessed using the maximum-likelihood (ML) method with MEGA12 software. The general time-reversible substitution model with gamma distribution (G) and invariant sites (I) (GTR + G + I) is suitable for the coding sequences of the three regions, namely polyprotein, MP, and CP. Branch support was evaluated by a bootstrap analysis using 1000 pseudoreplicates for the ML analyses. The resulting trees were then subjected to visualization in TreeView.

### 2.3. Nucleotide Compostion Analysis

Following the exclusion of five non-biased codons (UAA, UGA, UAG, termination codons) and the single-codon amino acids AUG (Met) and UGG (Trp), compositional parameters were calculated for the three CLBV protein-coding sequences. Overall nucleotide composition (A, C, U, and G%) and AU and GC content were determined in BioEdit. The nucleotide composition at the third codon position of the three CLBV protein-coding sequences (A3, C3, U3, and G3%) were calculated using the CodonW 1.4.2. The GC content at the 1st, 2nd, and 3rd codon positions (GC1, GC2, GC3), and the GC12 (the mean of GC1 and GC2) were computed by Python v3.13. The dinucleotide analysis was computed using the online platform EMBOSS (https://www.bioinformatics.nl/cgi-bin/emboss/compseq, accessed on 25 June 2025). Codon pair bias (CPB) refers to the non-random usage of synonymous codon pairs within protein-coding regions of genomes [25]. The codon pair bias (CPB) analysis was performed with Python v3.13.

### 2.4. Relative Synonymous Codon Usage (RSCU) Analysis

The RSCU is defined as the ratio of the observed frequency of a codon to the frequency expected under uniform synonymous codon usage [26]. It used to measure the degree of codon preference. RSCU values > 1.6 are considered over-represented for the more abundant codons, and <0.6 are regarded under-represented for the less abundant [27]. The calculation formula is as follows:

$${\mathrm{RSCU}}_{\mathrm{ij}}\;=\;\frac{g_{ij}}{\sum_{j}^{ni}g_{ij}}\times ni$$

RSCU_ij_ represents the value of the *i*th codon for the *j*th amino acid; whereas *g_ij_* is the cumulative count of all synonymous codons that encode the *j*th amino acid, and *ni* is the total number of synonymous codons that encode the *j*th amino acid. Python v3.13 was used to calculate the RSCU values of the CLBV three protein-coding sequences.

### 2.5. Effective Number of Codons (ENC) Analysis

The ENC is a quantitative metric that quantifies the degree of sequence codon preference. It ranges from 20, indicating an extreme preference for one synonymous codon per amino acid, to 61, indicating no preference and an equal use of synonymous codons per amino acid [28]. The ENC values were calculated via the EMBOSS online platform (https://emboss.bioinformatics.nl/cgi-bin/emboss/, accessed on 27 June 2025).

### 2.6. ENC-Plot Analysis and Selection Pressure Analysis

The ENC-plot was generated using R 4.5.0 studio, with the X-axis designated as GC3 and the Y-axis assigned to the ENC value. In the absence of selection, codon usage is dictated solely by mutational bias, and data dots fall on, or close to, the standard curve. Deviations below the curve indicate that natural selection, together with mutational pressure and other evolutionary forces, shapes codon bias patterns [29]. We performed a selection pressure analysis using the Branch-site Unrestricted Statistical Test for Episodic Diversification with Multiple Hits (BUSTED-MH) method on the Datamonkey platform (https://www.datamonkey.org/, accessed on 6 July 2025).

### 2.7. Parity Rule 2 Analysis

PR2-bias plots were employed to investigate the relative contributions of natural selection and mutation pressure on the codon usage of the CLBV, with G3/(G3 + C3) and A3/(A3 + T3) plotted on the abscissa and ordinate, respectively. When codon usage bias is solely driven by mutation pressure, A=T and G=C, with data dots locating at the midpoint (X = 0.5, Y = 0.5) [30]. The contents of A3, T3, G3, and C3 for each CLBV sequence were calculated and plotted using R 4.5.0 studio.

### 2.8. Neutrality Analysis

In the neutrality plot graph, GC12 and GC3 are shown as the ordinate and abscissa, respectively, to quantify the relative influences of mutation pressure, natural selection, and other factors on codon usage bias. In the absence of selection pressure, or if the selection pressure is weak, the slope of the regression line is near 1.0 [31]. Conversely, if the regression line slope deviates from 1.0, this indicates that natural selection is pivotal in codon bias [32]. The neutrality plots were generated in R 4.5.0 studio.

### 2.9. Codon Adaptation Index (CAI) Analysis

The CAI reflect the adaptability of individual viral genes to their hosts. The range of CAI is from 0 to 1, with higher values indicating closer codon usage matching and, consequently, stronger host adaptation [33]. The CAI values were calculated on the CAIcal online server (http://genomes.urv.cat/CAIcal, accessed on 9 July 2025) with host codon table data retrieved from the Codon Usage Database (http://www.kazusa.or.jp/codon, accessed on 12 July 2025). Following the grouping of the CAI values by host, the generation of visualizations was undertaken using R 4.5.0 studio.

## 3. Results

### 3.1. Recombination Analysis

Prior to downstream analyses, a rigorous screening for recombination events was conducted on the 56 CLBV genomes. This is an essential step because recombination has the capacity to distort both phylogenetic topology and genome-wide codon usage signatures. Following the identification and exclusion of ten isolates exhibiting clear recombinant signals, 46 non-recombinant genomes were selected for further analysis. The 46 CLBV remaining genome sequences included 18 from citrus, 26 from *Actinidia*, and 2 from *malus*. In the recombination events detected, breakpoints were predominantly localized within the CP region of CLBV, with sporadic events detected in the MP and the polyprotein regions. Notably, *Citrus* spp. constituted the major or minor parental lineage for the majority of these recombinant isolates (Table 1).

### 3.2. Phylogenetic Analysis

**Figure 1:**
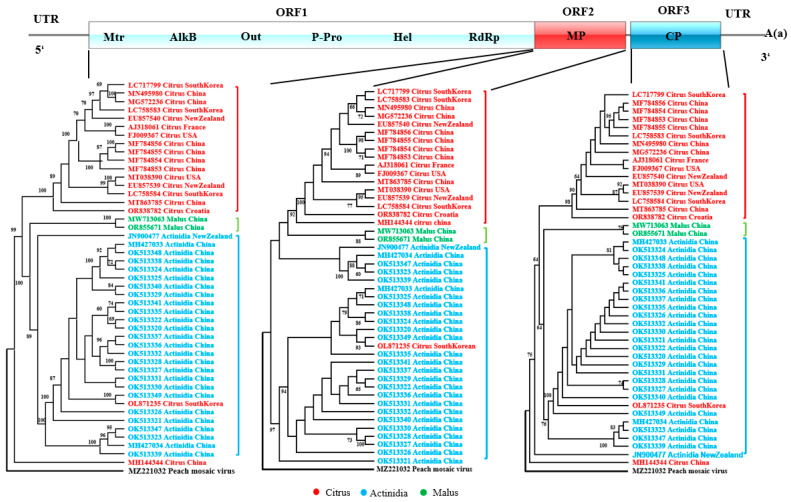
The maximum-likelihood (ML) tree calculated from the individual protein-coding sequences of CLBV.

Maximum-likelihood phylogenies were inferred separately for each of the three CLBV-encoded proteins. The results showed that the sequences exhibited pronounced host origin clustering. In particular, the polyprotein and CP-based trees revealed that all Actinidia-derived isolates formed a single, well-supported clade, the two malus-derived isolates constituted a second distinct clade, while the citrus-derived isolates were resolved into three separate lineages: one exclusive citrus clade, one nested within the Actinidia clade, and the remaining forming a second citrus-specific clade (Figure 1). These findings suggest that host adaptation has played a significant role in shaping viral diversification during the process of spread, and that host origin exerts a pronounced influence on sequence variation. Consequently, host origin was incorporated as a pivotal factor in all subsequent codon usage analyses.

### 3.3. Nucleotide Composition Analysis

The nucleotide compositions of the three protein-coding sequences of CLBV were assessed to explore the effect of compositional constraints on codon usage. Across the CLBV polyprotein region, adenine (30.8 ± 0.38%) and uracil (29.4 ± 0.33%) were the dominant nucleotides; the CP region displayed the same A/U bias, whereas the MP region showed A (32.79 ± 0.32%) and G (25.08 ± 0.34%) as the two most abundant bases. At the third codon position, the polyprotein and MP regions exhibited the hierarchy U_3_ > A_3_ > G_3_ > C_3_ (polyprotein: 33.03 ± 0.63%, 25.96 ± 0.96%, 20.99 ± 0.80%, 20.02 ± 0.67%, and MP), while the CP region followed A_3_ ≈ U_3_ > G_3_ > C_3_ (31.02 ± 1.78%, 30.57 ± 1.37%, 20.77 ± 1.16%, 17.64 ± 0.72%) (Table S2). In conclusion, the predominance of A/U was observed at all three codon positions. The composition of A/U is superior to the GC of three protein-coding sequences, suggesting that A/U is more prevalent in the CLBV coding sequences. The results of the dinucleotide analysis and codon pair bias (CPB) are provided in Supplementary Table S4, respectively. Our dinucleotide analysis revealed a genome-wide depletion of CG dinucleotides and an enrichment of AU/UA dinucleotides in the CLBV genome. Subsequent codon pair analyses further corroborated this trend.

### 3.4. RSCU Analysis of CLBV

**Figure 2:**
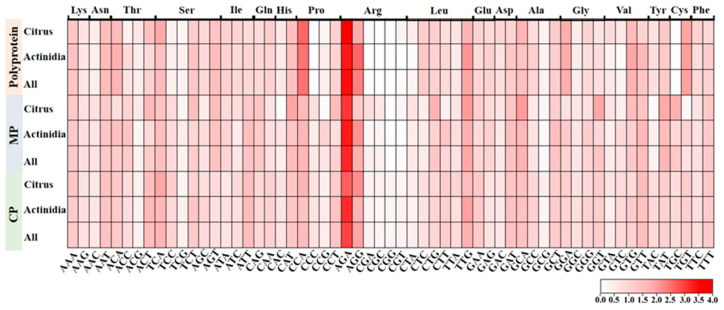
RSCU heatmap of 59 codons encoding 18 amino acids across 3 CLBV proteins.

In order to estimate the codon usage pattern of the protein-coding sequences of CLBV, the RSCU values were first calculated. The CLBV protein-coding sequence exhibited marked codon usage biases for multiple amino acids, including arginine (Arg), asparagine (Asn), histidine (His), leucine (Leu), threonine (Thr), and serine (Ser). For instance, Arg is preferentially encoded by AGA and AGG, with the RSCU value of AGA ranging from 2.65 to 3.66 across different hosts and coding regions, while the codons CGA, CGT, CGC, and CGG are strongly avoided. Furthermore, it was observed that most high-frequency codons favored termination with A/T, while a majority of low-frequency codons favored to end with G/C. Simultaneously, we identified pronounced host-dependent differences in codon usage within specific coding regions. Specifically, cysteine (Cys) is preferentially encoded by TGC in the polyprotein-coding region in citrus hosts (with TGT disfavored), whereas the opposite pattern-strong preference for TGT and avoidance of TGC is observed in *Actinidia* hosts (Figure 2, Table S3).

### 3.5. Codon Usage Bias of CLBV

**Figure 3:**
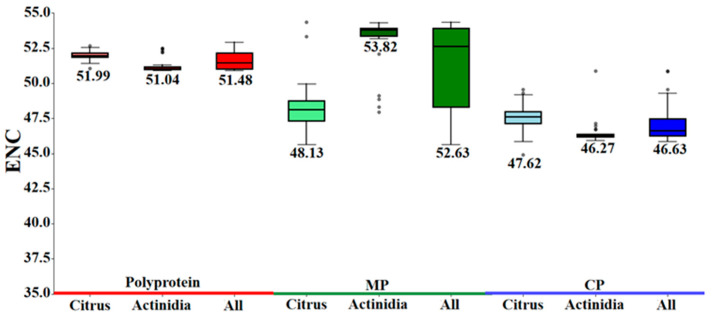
ENC values for the three coding sequences of CLBV.

The effective number of codons (ENC) is a metric of codon usage bias that is both length and composition independent. The scale ranges from 20 (extreme bias) to 61 (no bias); values < 35 denote strong bias, whereas those > 55 indicate weak bias [28]. Across the three CLBV protein-coding regions, ENC varied narrowly (47.06–51.63), reflecting a globally conserved nucleotide composition and low overall bias. Nevertheless, host-specific divergence was evident: the MP region exhibited the most pronounced shift, with ENC values of 48.55 in *Citrus* and 52.94 in *Actinidia*, implying adaptive modulation of codon usage during host specialization (Figure 3).

### 3.6. ENC-Plot and Neutrality Analysis

**Figure 4:**
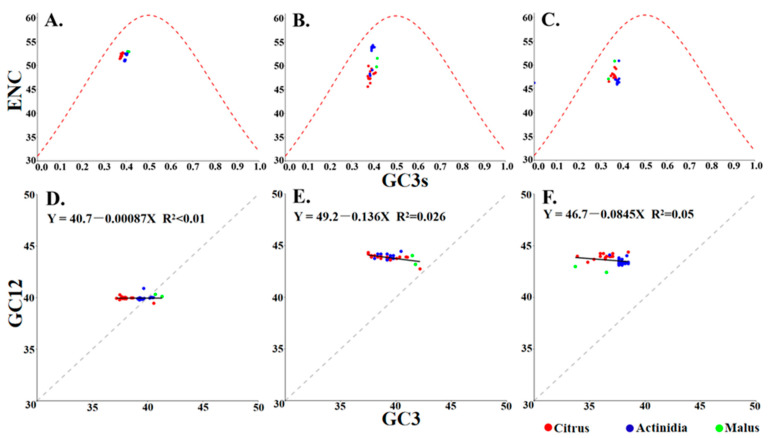
ENC-plot and neutrality analysis of the individual protein-coding sequences of CLBV. (**A**–**C**) ENC-plots for polyprotein, MP, and CP; (**D**–**F**) Neutrality plots for polyprotein, MP, and CP.

ENC-plot and neutrality analysis jointly dissect the evolutionary forces shaping codon usage by contrasting observed ENC or GC_12_–GC_3_ relationships with their mutational expectations. This approach enables the quantification of the relative contributions of natural selection and mutational bias. It is hypothesized that under conditions of pure mutational pressure, ENC-plot points will be expected to lie on the theoretical curve, and that the neutrality plot will yield a regression slope of 1. In the ENC-plot generated for the three protein-coding sequences of CLBV, all data points representing CLBV isolates from different hosts cluster below the expected curve. This finding suggests that natural selection dominated over mutation pressure, while the influence of mutation was not completely absent. Consistently, neutrality analysis showed that no significant correlation was observed between the GC12 and GC3 for the three protein-coding sequences of CLBV. The slope of the linear regression for protein-coding sequences was found to be close to zero, indicating that codon usage is predominantly governed by natural selection. However, the slopes differed among the coding regions. The regression slope for the MP coding region was found to be −0.136, indicating a mutation pressure of 14.6% and a natural selection effect of 85.4%, representing the weakest natural selection among the three coding regions (Figure 4). The results obtained collectively indicated that natural selection is the principal driving force for the formation of codon usage bias in CLBV. The detailed data from the selection pressure analysis are provided in Supplementary Table S4. The selection pressure analysis results revealed that significant episodic positive selection was detected in both the CP and polyprotein genes: In the CP gene, 99.754% of sites were under strong negative selection (dN/dS = 0.044/0.048), while 0.246% of sites experienced extremely strong positive selection (dN/dS = 65.717). In the polyprotein gene, 99.947% of sites were under negative selection (dN/dS = 0.001/0.207), whereas 0.053% of sites were under strong positive selection (dN/dS = 50.510). These findings provide supporting evidence for the view that natural selection influences the formation of codon usage bias in CLBV.

### 3.7. PR2 Analysis

**Figure 5:**
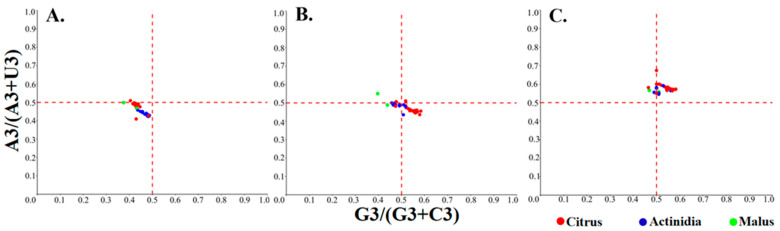
Parity Rule 2 analysis of the individual protein-coding sequences of CLBV. (**A**–**C**) PR2 plots for polyprotein, MP, and CP.

In the PR2-bias plot, the absence of both directional mutation pressure and natural selection is represented by the intersection of the two reference lines (X = 0.5 and Y = 0.5), where the frequencies of G and C, as well as A and T, are equal. In the CP region, all points exhibited Y values > 0.5, indicating a consistent bias toward A over T. Most citrus samples exhibited a clustering in the quadrant where X values exceeded 0.5, thereby revealing a pronounced preference for G over C, whereas Acitinidia-derived samples were positioned close to X = 0.5, denoting nearly equal usage of G and C. Within the MP region, the majority of points fell below Y = 0.5, thus signifying a clear bias towards T relative to A. Citrus samples once again displayed a G-biased pattern (X > 0.5), whereas Acitinidia samples showed a C-biased pattern (X < 0.5). In the polyprotein region, most points were located in the quadrant where both X and Y values were less than 0.5, indicating a simultaneous preference for T and C over their complementary nucleotides (Figure 5). Collectively, these findings revealed markedly heterogeneous nucleotide preferences among coding regions, with the same region displaying host-specific GC/AT skews in citrus versus Actinidia-infecting isolates. This implies that viral genome evolution is jointly shaped by the functional constraints and host environment.

### 3.8. CAI Analysis of Sequences to Different Hosts

**Figure 6:**
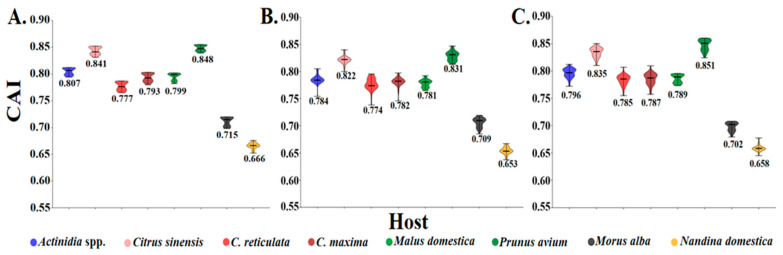
The codon adaptation index (CAI) analysis of the individual protein-coding sequences of CLBV. (**A**–**C**) The plots for polyprotein, MP, and CP.

To quantify the adaptation and codon usage optimization of CLBV to its hosts, codon adaptation index (CAI) values were calculated. It is generally accepted that higher CAI values are indicative of stronger translational adaptation to the host tRNA pool, and that this, in turn, leads to superior host compatibility. The highest average CAI values were identified in Prunus avium (0.828–0.848) and the lowest were identified in Nandina domestica (0.654–0.666), indicating that among the documented hosts, Prunus avium represents the most suitable host, whereas Nandina as the least favorable (Figure 6). Consistently, among citrus cultivars, Citrus sinensis exhibited the highest CAI values, thereby further suggesting that Citrus sinensis is the most suitable citrus host for CLBV. Nevertheless, the current number of full genome sequences for certain hosts remains limited, which may introduce bias into the CAI estimation. Consequently, the incorporation of additional genome sequences will be essential to obtain a more robust and representative assessment.

## 4. Discussion

The present study utilized a genome-wide analysis of 46 non-recombinant CLBV to uncover that the virus evolution is predominantly shaped by host-specific translational selection. Despite the globally low codon usage bias, synonymous codon choice is stratified by host taxonomy rather than geography, phylogenies across all three ORFs recapitulate host lineage, and recombination breakpoints are disproportionately enriched in the coat protein gene, the very locus where host-determined codon signatures peak. Collectively, these data imply natural selection for tRNA compatibility as a major driver of CLBV divergence, with RNA recombination serving as an expedient route to import pre-adapted, host-optimized modules.

### 4.1. Recombinant Event Analysis

We detected ten unique recombination events, 80% of which map to the CP cistron and involve citrus-derived parental lineages. This breakpoint preference is unlikely to be stochastic: the CP is the interface between virion and host chaperones during cell-to-cell movement, and its surface-exposed residues are subject to strong selective sweeps. The AU-richness and predicted stem-loop structures within this region may promote RdRp template switching via the copy-choice mechanism [34,35], as documented in other flexiviruses. By shuffling CP modules that have already been fine-tuned for citrus translation machinery, CLBV can instantaneously increase fitness in new hosts, a strategy reminiscent of TYLCV recombinants that expanded their tropism to common genes [36,37]. Consequently, recombination appears to act as an “evolutionary shortcut”, allowing the virus to sample pre-adapted sequences without the slow accumulation of point mutations.

### 4.2. Host-Driven Divergence Predominates over Geographic Structuring in CLBV Phylogeny

CLBV phylogenies exhibit pronounced host fidelity: *Actinidia* spp. isolates form a robust monophyletic clade, whereas citrus-derived sequences are dispersed across several lineages, mirroring the host-associated topology reported for CABYV [38]. This host-first, geography-second structure implicates adaptation to divergent host translational environments as the primary driver of CLBV divergence, a scenario reinforced by the parallel host-specific codon usage split most strikingly seen in the movement protein gene. Natural selection, not spatial isolation, is therefore the dominant force sculpting the genome of this generalist virus.

### 4.3. Genome-Wide and Gene-Specific Nucleotide Signatures Reflect Host-Driven Selection in CLBV

The genome of CLBV exhibits a pronounced nucleotide bias, with significant enrichment of adenine (A) and uracil (U) at multiple sites and a near-complete exclusion of CpG-containing CGN codons. This tactic is not unique: a substantial fraction of plant viruses also favor A-rich sequences [39,40], a preference driven by convergent evolutionary pressures including immune evasion, replication efficiency, translational optimization, and long-term adaptive selection [41,42,43,44]. Strikingly, the A-rich, C-depleted signature of CLBV mirrors the CpG-suppressed profiles documented in animal RNA viruses, implying that both plant and animal pathogens exploit analogous ‘linguistic camouflage’ to evade conserved antiviral defenses [45]. Discovered in animals, Zinc Finger Antiviral Protein (ZAP) recognizes CpG dinucleotides in viral RNA through its N-terminal RNA-binding domain containing four CCCH-type zinc fingers and a specific binding pocket, and distinguishes non-self RNA based on the CpG suppression feature of the host genome. After binding to CpG, ZAP forms a complex with TRIM25, recruits nucleases to degrade viral RNA, and may also indirectly activate the interferon pathway, thereby triggering the host’s immune response. In order to circumvent the triggering of the host’s immune response, animal RNA viruses have evolved to reduce CpG content [46,47].

Beyond genome-wide compositional bias, CLBV also displays gene-specific codon usage patterns. Within the polyprotein region of citrus-adapted isolates, cysteine is almost exclusively encoded by TGT (RSCU = 1.94), whereas TGC is strongly avoided (RSCU = 0.06). Conversely, the MP region exhibits the opposite preference: TGC is favored (RSCU = 1.82) and TGT is disfavored (RSCU = 0.18). These reciprocal biases corroborate earlier reports that distinct genes within a single viral genome can experience different translational or selective environments [48,49].

There is a large difference in ENC values between the CP and MP genes of CLBV in Actinidia hosts. A similar pattern has been reported for the Usutu virus, where the ENC of the NS4A gene differs markedly from those of NS2B and NS4B. This discrepancy arises because the different genes of the Usutu virus experience distinct equilibria between selective and mutational pressures during evolution; for this flavivirus, codon usage is governed primarily by natural selection rather than by mutation pressure [50]. Analogously, codon usage in CLBV is also dominated by natural selection with only minor influence from mutation pressure, giving rise to the same inter-gene heterogeneity in ENC values.

### 4.4. Codon Adaptation Index

From the perspective of the CAI, *Prunus avium* appears to be the most adapted host, while *Nandina domestica* is the least adapted. A higher CAI is indicative of tighter adaptation to a host and, by extension, a potentially longer co-evolutionary history. Consequently, the hypothesis can be formulated that *P. avium* represents an ancestral rather than a recently acquired host of CLBV. However, given that the virus was initially reported in citrus and subsequently described in *Actinidia*, the majority of existing studies, and the corresponding genomic datasets have focused exclusively on these two hosts, with few studies focusing on cherry trees. Therefore, comprehensive investigations into the prevalence, geographic distribution, and genomic characteristics of CLBV infecting cherry trees are urgently warranted. Meanwhile, the question of whether superior host adaptation translates into higher viral accumulation and, consequently, more pronounced symptoms remains an open question that requires systematic experimental validation in future studies. A parallel question has been raised in the context of citrus: CAI analyses indicate that CLBV is more adapted to *Citrus sinensis* than to other citrus cultivars. Whether this enhanced codon adaptation corresponds to higher viral titres or more severe symptoms in *Citrus sinensis* remains to be empirically tested.

## 5. Conclusions

By integrating full-genome phylogenetics with multilayer codon usage analyses, we show that host-driven translational selection, rather than geographic distance, play a major role in the molecular evolution of CLBV. Despite an overall low codon usage bias, CLBV exhibits host-specific nucleotide and codon signatures that are strongest in the coat protein gene, a region that has been identified as a recombination hotspot. The virus achieves optimal adaptation in *Prunus avium* (highest CAI) and undergoes accelerated evolution in *Nandina domestica* (lowest CAI), indicating that synonymous site evolution is a reliable proxy for host compatibility. Natural selection, reinforced by modular RNA recombination, thus provides CLBV with a rapid pathway to fine-tune its translational landscape across divergent hosts. These findings establish a genome-wide baseline for the monitoring of emerging CLBV lineages and inform the design of host-adapted, codon-optimized viral vectors for sustainable pomiculture.

## Figures and Tables

**Table 1:** Recombination events detected in CLBV full-length genomes.

Recombinant Isolates	Major Parent	Minor Parent	Breaking Point Position (bp)	Coding Protein
***Nandina*** (MT078932)	*Malus* (MW713062)	*Citrus* (OL871235)	7008–8786	MP, CP
***Morus*** (MT767171)	*Actinidia* (OK513339)	*Citrus* (OL871235)	7274–8161	MP, CP
***Actinidia*** (MG604237)	*Actinidia* (OK513347)	*Actinidia* (OK513324)	1–2229	Polyprotein
***Prunus*** (KR023647)	*Citrus* (LC758583)	*Malus* (MW713062)	7567–8762	CP
***Morus*** (OP971103)	*Malus* (MW713062)	*Citrus* (AJ318061)	7280–8617	CP
***Viburnum*** (OP751940)	*Citrus* (OR838782)	*Actinidia* (OK513336)	8246–8817	CP
***Malus*** (MW713062)	*Actinidia* (OK513339)	*Citrus* (OL871235)	7259–8491	CP
***Actinidia*** (MK135436)	*Nandina* (MT078932)	*Actinidia* (JN900477)	6889–8678	MP, CP
***Citrus*** (MH558590)	*Actinidia* (OK513326)	*Citrus* (LC758583)	6279–6577	MP, CP
***Malus*** (OR855671)	*Citrus* (OR838782)	*Viburnum* (OP751940)	8205–8787	CP

**Note: The parentheses contain the GenBank accession numbers of the sequences.**

## Data Availability

All data are presented in the manuscript and the Supplementary Materials.

## Supplementary Materials

The following supporting information can be downloaded at: https://www.mdpi.com/article/10.3390/v17111497/s1, Table S1: Metadata for the CLBV isolates used in this study; Table S2: Details of the nucleotide composition at synonymous third codon positions of CLBV coding sequences; Table S3: RSCU values of CLBV coding regions across host-associated sequence sets; Table S4: Dinucleotide analysis of CLBV coding sequences.
